# TRIM5 inhibits the replication of Senecavirus A by promoting the RIG-I-mediated type I interferon antiviral response

**DOI:** 10.1186/s13567-024-01354-2

**Published:** 2024-08-14

**Authors:** Huizi Li, Zhenxin Xie, Xiaoling Lei, Ming Chen, Tingting Zheng, Cunhao Lin, Zhangyong Ning

**Affiliations:** 1 College of Veterinary Medicine, South China Agricultural University, Guangzhou, 510642 China; 2 Maoming Branch, Guangdong Laboratory for Lingnan Modern Agriculture, Maoming, 525000 China

**Keywords:** Senecavirus A, TRIM5, RIG-I, IFNs, inflammatory cytokines

## Abstract

Senecavirus A (SVA) is an emerging virus that poses a threat to swine herds worldwide. To date, the role of tripartite motif 5 (TRIM5) in the replication of viruses has not been evaluated. Here, TRIM5 was reported to inhibit SVA replication by promoting the type I interferon (IFN) antiviral response mediated by retinoic acid-inducible gene I (RIG-I). TRIM5 expression was significantly upregulated in SVA-infected cells, and TRIM5 overexpression inhibited viral replication and promoted IFN-α, IFN-β, interleukin-1beta (IL-1β), IL-6, and IL-18 expression. Conversely, interfering with the expression of TRIM5 had the opposite effect. Viral adsorption and entry assays showed that TRIM5 did not affect the adsorption of SVA but inhibited its entry. In addition, TRIM5 promoted the expression of RIG-I and RIG-I-mediated IFNs and proinflammatory cytokines, and this effect was also proven by inhibiting the expression of TRIM5. These findings expand the scope of knowledge on host factors inhibiting the replication of SVA and indicate that targeting TRIM5 may aid in the development of new agents against SVA.

## Introduction

Senecavirus A (SVA) is a non-enveloped, single-strand and positive-sense RNA virus that is the only member of the genus *Senecavirus* in the family *Picornaviridae* [1]. The virus induces porcine idiopathic vesicular disease (PIVD), which typically manifests as claudication and vesicular lesions in the oral area and on the snout and coronary bands. Since 2015, PIVD induced by SVA has been reported in major pig-raising countries, including the United States of America [2], Colombia [3], Thailand [4], Vietnam [5], and China [6]. The genome of the virus is approximately 7.3 kb in length and contains four structural proteins (VP1, VP2, VP3, and VP4) and eight nonstructural proteins (L, 2 A, 2B, 2 C, 3 A, 3B, 3 C, and 3D) [7]. The virulence of the epidemic strains in recent years has increased compared to that of historical strains due to the basic characteristics of rapid evolution and continuous recombination, which increase the difficulty of preventing and controlling this viral infection [8].

Recent reports have shown that SVA can establish persistent infection in pigs and become a source of infection [9], indicating that SVA and its encoded proteins have developed a variety of tactics to escape the host’s innate immune response, including reducing the production of interferons (IFNs) [10, 11], inducing selective apoptosis [12], and activating autophagy, which promotes virus proliferation [13]. Although the impacts of the virus on host cells are relatively clear, the effects of host factors on the virus are not well understood. Some host proteins have been shown to impact SVA proliferation. Myxoma resistance 1 (Mx1) inhibits SVA replication [14], interferon-induced protein with tetratricopeptide repeats 3 (IFIT3) suppresses SVA replication by regulating the antiviral response via IFNs [15], and suppressor of cytokine signalling 1 (SOCS1) and mitofusin-2 (Mfn2) restrain the antiviral response, facilitating SVA infection [7, 16].

The tripartite motif (TRIM) family has been identified as potential virus-limiting factors that exhibit E3 ubiquitin ligase activity, which plays an important role in innate antiviral immunity [17]. These proteins are involved in the regulation of various physiological and pathological processes, such as intracellular signalling pathways, apoptosis, autophagy, tumorigenesis, and the innate immune response [18, 19]. TRIM5, which has multiple isoforms of protein products due to differential splicing of primary transcripts, is an important virus restriction factor that impedes the accumulation of reverse transcripts of HIV-1 in the early and late phases of the retroviral replication cycle by identifying and combining capsid through the PRYSPRY domain [20]. It has been reported that TRIM5α can recognize the capsid structure of retroviruses, promote premature uncoating of the capsid, and hinder retroviral replication [21]. TRIM5α inhibits human immunodeficiency virus (HIV) replication by binding to the HIV protease NS2B/3, promoting K48 ubiquitination and the proteasomal degradation of NS2B/3 [22]. TRIM5α also functions as a signal transducer to initiate intracellular innate immune responses by activating the nuclear factor-kappa B (NF-κB) and activating protein-1 (AP-1) signalling pathways [23, 24]. Although the function of TRIM5 has been preliminarily clarified, its role in the replication of SVA has not been identified.

Here, we report that porcine TRIM5 inhibits SVA replication by promoting the type I IFN antiviral response mediated by retinoic acid-inducible gene I (RIG-I). This provides new data on the role of host factors in the replication of SVA and indicates that targeting TRIM5 may help to develop new agents to counter infection and replication of the virus.

## Materials and methods

### Viruses, cells, and viral infection

The SVA strain CH-GDFS-2018 was isolated and identified in 2018 [25]. The virus was propagated and titrated in porcine alveolar macrophages (3D4/21) and porcine kidney-15 (PK-15) cells using the Reed and Muench method [7]. 3D4/21 and PK-15 cells were cultured with 1640 complete (Gibco, USA) and Dulbecco’s modified Eagle’s medium (Gibco, USA), respectively, supplemented with 10% foetal bovine serum (FBS) (Gibco, USA), 100  IU/mL penicillin, 100 μg/mL streptomycin, and 2 mM L-glutamine and cultured at 37 °C and 5% CO_2_. Cells were cultured in 6-well plates and infected with SVA at a multiplicity of infection (MOI) of 1. In the adsorption assay, cells were infected with SVA at an MOI of 1 at 24 h after transfection and incubated at 4 °C for 1 h. The unbound virions were washed off with ice-cold precooled phosphate-buffered saline (PBS) three times, and the cell samples were collected for total RNA extraction. In the entry assay, preheated maintenance medium containing 2% FBS was added to the cells after infection with SVA, and the cells were incubated at 4 °C for 1 h. The cells were incubated at 37 °C with 5% CO_2_ for 2 h, after which the cell samples were collected.

### Overexpression and RNA interference

The primers F (5′-CCG*CTCGAGG*CCACCATGGCTTCAGGCATCCTGGA-3′) and R (5′-CCG*GAATTCG*AGAGCCCGGCGAGCACAGA-3′), which contain the *Xho *I and *Eco*R I restriction sites, respectively, were designed based on porcine TRIM5 with the GenBank accession number NM_001044532 and synthesized by Sangon Biotech (Shanghai, China). The sequence encoding TRIM5 was obtained from the cDNA of 3D4/21 cells and subcloned and inserted into the vector pEGFP-N1 (Clontech, USA) to construct a pEGFP-TRIM5 overexpression plasmid. The specific target sequences shTRIM5 (5′-GCGCTTTCTGCTCAAGAATCT-3′), shRIG-I (5′-GCCCTTAACCAAGCAGGTTAT-3′), and shNC (negative control) (5′-GGACGAGGAGAAAGTTATTCT-3′) were designed and synthesized by Sangon Biotech (Shanghai, China) and cloned and inserted into the pSuper.neo RNAi plasmid (Oligoengine). Lipofectamine 2000 reagent was used to transfect the plasmid into 3D4/21 cells grown in a 6-well plate according to the manufacturer’s instructions (Invitrogen, USA).

### Preparation of anti-porcine TRIM5 polyclonal antibodies

The primers F (5′-CCG*GAATTC*GCCACCATGGCTTCAGGCATCCTGGA-3′) and R (CCG*CTCGAG*TAGAGCCCGGCGAGCACAGAA), which contain the *Eco*R I and  I restriction sites, respectively, were designed and synthesized by Sangon Biotech (Shanghai, China). The TRIM5-His fusion protein was induced by isopropyl β-d-1-thiogalactopyranoside (IPTG), and the antiserum preparation and IgG purification of the TRIM5 proteins were carried out according to the methods of our laboratory [26]. After immunization, the titres of the rabbit antisera were determined by enzyme-linked immunosorbent assay (ELISA) [26].

### RNA extraction and quantitative real-time PCR (qRT-PCR).

Total RNA was extracted at the indicated time points using TRIzol reagent (Invitrogen, USA) and reverse transcribed to cDNA using a reverse transcription kit (Takara, Japan) according to the manufacturer’s instructions. cDNA was amplified with SYBR Green Pro Taq HS Premix (Accurate, China) in a Light Cycler 480 (Roche, Switzerland). The primers used for qRT-PCR are shown in Table 1. The relative expression of the target genes was calculated using the 2^−ΔΔCt^ method [7].

**Table 1:** **Primer sequences for qRT-PCR**

Target Gene	Sequence (5′–3′)
GAPDH	F: AAGGGCATCCTGGGCTACACTGAG
	R: TGAGGTCCACCACCCTGTTGCTGT
TRIM5	F: GGAGACAAGTGAGATACGCGC
	R: CCTACCATACACCCCCAAGAT
SVA-VP3	F: AACCGACCTCTTACAACTGG
	R: CTTGCCATCATAGGTCCACA
IFN-α	F: CTGGCACAAATGAGGAGAAT
	R: TGCTGAAGAGCTGGAAGGT
IFN-β	F: CTCTAGCACTGGCTGGAATGAA
	R: CCGGAGGTAATCTGTAAGTCTGTT
RIG-I	F: CTCGGTGGCAGATGAAGG
	R: AGCGTTAGCAGTCAGAAGG
MAVS	F: AGAAGCAGGACACAGAAC
	R: GAGGAGGAGGCAGTAGAC
IRF3	F: GCAGGAGGACTTCGGCATCT
	R: TGGGCAGGTCGGGCTTAT
IRF7	F: GACTTCGGCACCTTCTTCCA
	R: ATGGCTCCAACTTCACCAGG
TAK1	F: AGGTTGTTGGAAGAGGAGCC
	R: GCCCCCTTCAGCATATTCCA
IL-1β	F: CGTGCAATGATGACTTTGTCTGT
	R: AGAGCCTTCAGCATGTGTGG
IL-6	F: TGGCTACTGCCTTCCCTACC
	R: CAGAGATTTTGCCGAGGATGT
IL-18	F: GAACCTAAACTCTCAATCATACG
	R: ATAATAAATACGGTCTGAGGTGC

### Western blot

The cell samples were lysed with radio immunoprecipitation assay (RIPA) lysis buffer (DGCS Biotechnology, China), and the proteins were collected via centrifugation at 12000 × *g* for 15 min at 4 °C. The proteins were detected using anti-β-actin (Boiss, China), anti-EGFP (Boiss, China), anti-RIG-I (Boiss, China), and anti-VP2 antibodies according to our previous research [7].

### Statistical analysis

GraphPad Prism version 8.0 software (GraphPad Software, La Jolla, CA, USA) was used for *t* tests and one-way analysis of variance. The data are expressed as the mean ± standard deviation (SD) from at least three independent experiments. A value of *P* < 0.05 compared with the control was considered significant.

## Results

### Antiserum preparation and IgG purification

**Figure 1:**
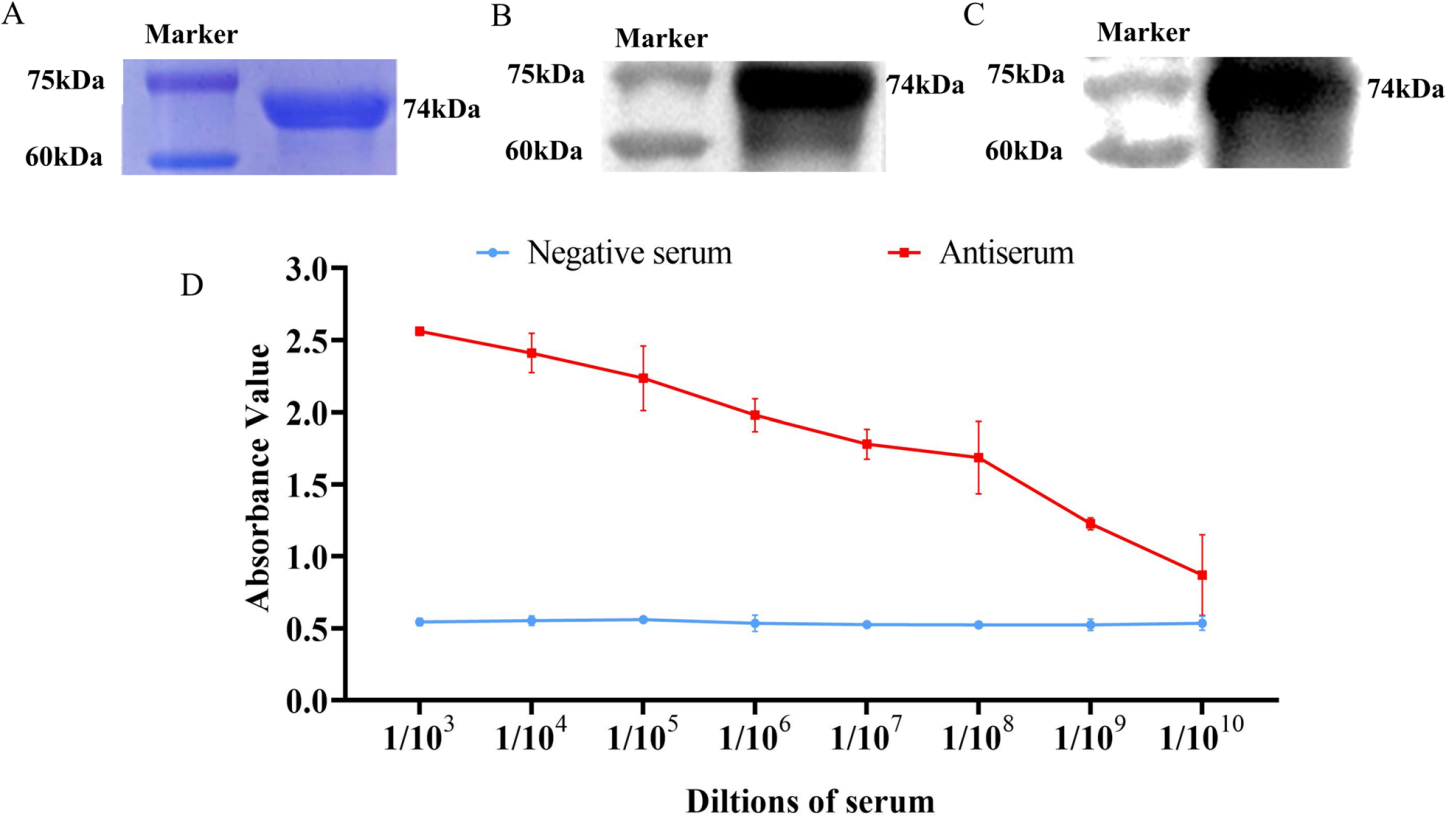
**Antiserum preparation and IgG purification. A** The purified recombinant His-TRIM5 was detected by SDS-PAGE. **B** The purified recombinant protein was detected by western blotting with rabbit anti-His-tag antibody as the primary antibody. **C** The purified recombinant proteins were detected by western blotting with prepared anti-pTRIM5 IgG as the primary antibody. **D** The titre of the serum was detected using ELISA.

Commercial anti-TRIM5 antibodies are available only for humans, while the homology of TRIM5 proteins between humans and swine is only 56.5%. An anti-porcine-TRIM5 (anti-pTRIM5) antibody was used for protein detection in subsequent experiments. The SDS‒PAGE results showed that the size of the purified His-TRIM5 protein band was consistent with the predicted size of approximately 74 kDa (Figure 1A). Western blotting showed that recombinant His-TRIM5 could be detected using an anti-His-tag antibody, indicating that prokaryotic protein expression was successful (Figure 1B). The recombinant protein was correctly detected by western blotting using prepared IgG as the primary antibody, and the results indicated that the prepared rabbit anti-pTRIM5 IgG had favourable specificity (Figure 1C). ELISA revealed the excellent potency of the anti-pTRIM5 serum at dilutions up to 1:10^9^ (Figure 1D).

### SVA infection increases the expression of TRIM5 in 3D4/21 cells

**Figure 2:**
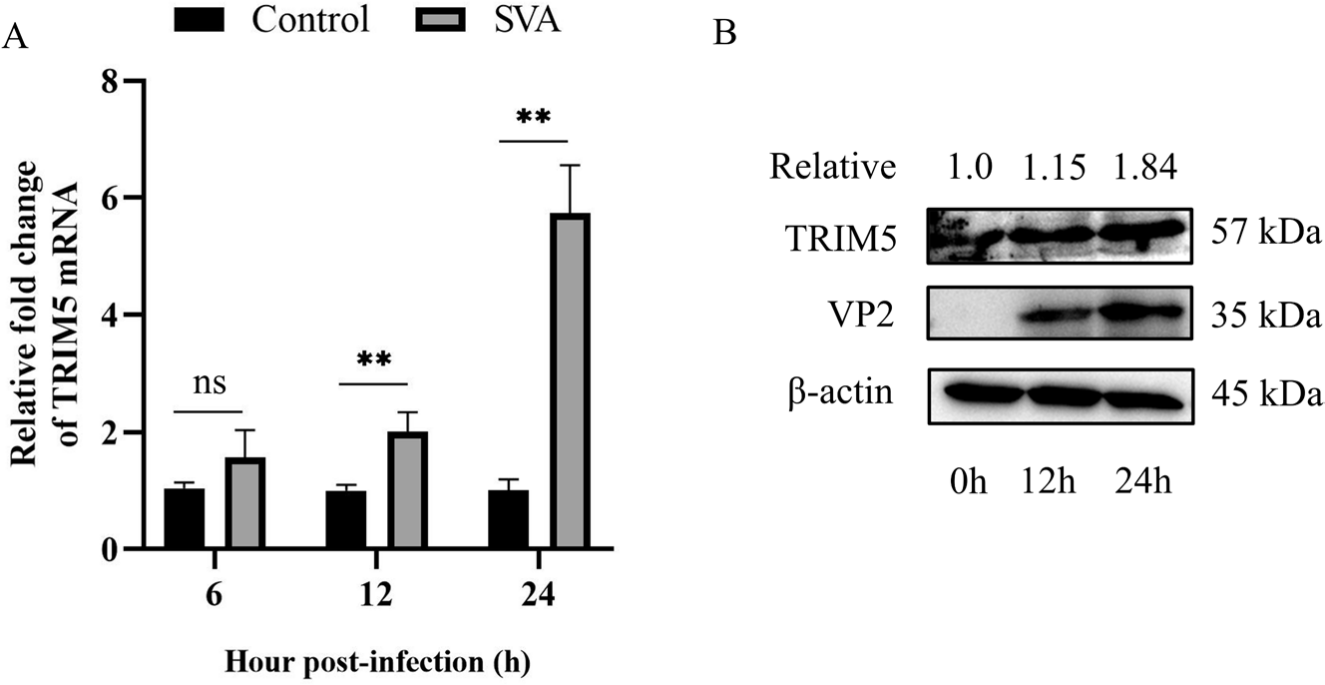
**SVA infection increases the expression of TRIM5 in 3D4/21 cells.** 3D4/21 cells were infected with SVA at an MOI of 1 in the test group but not in the control. **A** Cell samples were collected at 6, 12, and 24 hpi, and the mRNA expression of TRIM5 was analysed by qRT-PCR. **B** The protein expression levels of TRIM5 and SVA VP2 were detected by western blotting at 0, 12, and 24 hpi, and the relative mean expression of the TRIM5 protein was calculated by ImageJ. All experiments were repeated at least three times independently. ^*^P < 0.05, ^**^P < 0.01, ns indicates no significant difference.

To explore whether SVA infection affects TRIM5, 3D4/21 cells were infected with SVA in the test group but not in the control. The qRT-PCR results showed that the mRNA expression of TRIM5 in 3D4/21 cells infected with SVA was significantly greater than that in control cells at 12 and 24 h post-infection (hpi) (*P* < 0.01) (Figure 2A). Western blotting revealed that SVA infection increased the protein expression of TRIM5 at 12 and 24 hpi, which was positively correlated with the expression of SVA VP2 (Figure 2B).

### TRIM5 inhibits SVA replication in 3D4/21 cells

**Figure 3:**
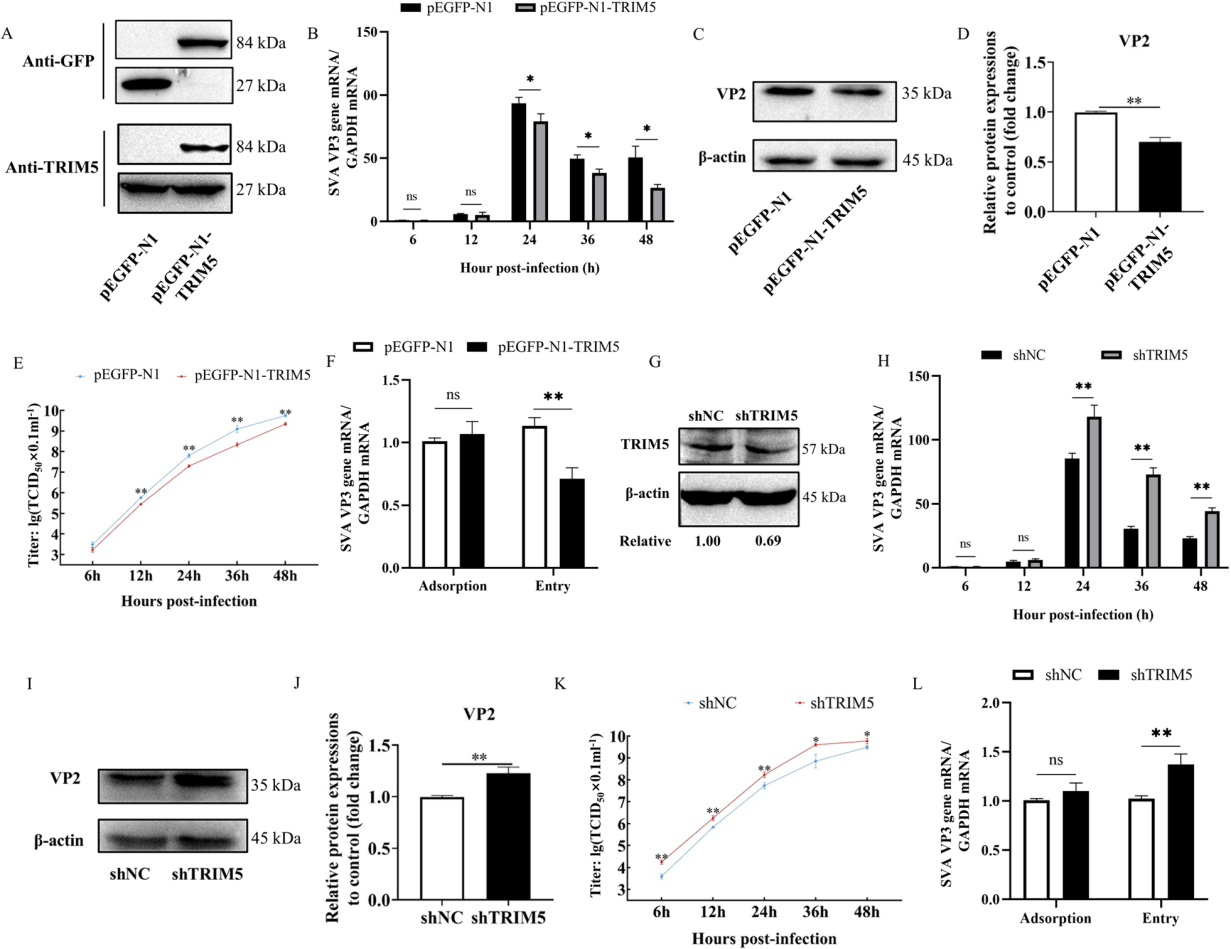
**TRIM5 inhibits SVA replication.** **A** 3D4/21 cells were transfected with pEGFP-N1 or pEGFP-N1-TRIM5 for 24 h, and the expression of TRIM5 was detected by western blotting. 3D4/21 cells were infected with SVA at an MOI of 1. **B** The expression of the SVA VP3 gene at 6, 12, 24, 36, and 48 hpi was analysed by qRT-PCR. **C**,** D** The protein expression of VP2 at 24 hpi was detected by western blotting and quantitatively analysed by Image J.** E** Viral titres were determined using TCID_50_ in PK-15 cells. **F** The expression of the SVA VP3 gene in adsorption and entry experiments was measured by qRT-PCR. 3D4/21 cells were transfected with shNC or shTRIM5 for 24 h. **G** The effectiveness of shTRIM5 was detected by western blotting. 3D4/21 cells were infected with SVA at an MOI of 1. **H** The mRNA expression of the SVA VP3 gene at 6, 12, 24, 36, and 48 hpi was analysed by qRT-PCR. **I**,** J** The protein expression levels of VP2 at 24 hpi were detected and analysed. **K** The viral titres were determined using TCID_50_ in PK-15 cells. **L** The expression of the SVA VP3 gene in adsorption and entry experiments was measured by qRT-PCR. All experiments were repeated at least three times independently. ^*^P < 0.05, ^**^P < 0.01, ns indicates no significant difference.

To determine the effect of TRIM5 on SVA proliferation, 3D4/21 cells were infected with SVA after overexpression or inhibited expression of TRIM5. The plasmid pEGFP-N1-TRIM5 was transfected into 3D4/21 cells, and the fusion protein GFP-TRIM5 was strongly expressed (Figure 3A). Compared with that in the control cells, the mRNA expression of the SVA VP3 gene in the viral genome in the pEGFP-N1-TRIM5-transfected cells decreased significantly from 24 to 48 hpi (*P* < 0.05) (Figure 3B). Compared with that in the control group, the expression of the VP2 protein in the pEGFP-N1-TRIM5 group was inhibited at 24 hpi (*P* < 0.01), which was the same trend as that observed for the mRNA levels (Figure 3C and D). The results of the 50% tissue culture infective dose (TCID_50_) assay showed that the virus titre in the pEGFP-N1-TRIM5 group was significantly lower from 12 to 48 hpi and approximately 10-fold lower at 36 hpi than that in the control group (*P* < 0.01) (Figure 3E). The results of adsorption and entry assays showed that the overexpression of TRIM5 did not affect the adsorption of SVA but inhibited its entry (*P* < 0.01) (Figure 3F). Experiments examining the interference of TRIM5 expression showed that the expression of the TRIM5 protein was 0.69 (Figure 3G). Viral infection experiments in cells with impeded TRIM5 expression showed that the mRNA expression of the SVA VP3 gene was significantly increased from 24 to 48 hpi compared to that in the control (*P* < 0.01) (Figure 3H), and the expression of the SVA VP2 protein was significantly upregulated at 24 hpi (*P* < 0.01) (Figure 3I and J). Analysis of the TCID_50_ showed that the virus titre in the shTRIM5 group was significantly greater than that in the control group at 6, 12, 24 (*P* < 0.01), 36, and 48 hpi (*P* < 0.05) and approximately 10-fold greater at 36 hpi (Figure 3K). Adsorption and entry assays showed that interfering with the expression of TRIM5 did not affect the adsorption of SVA but promoted its entry (*P* < 0.01) (Figure 3L). These results indicate that TRIM5 inhibits SVA replication.

### TRIM5 increases the expression of IFNs and proinflammatory cytokines in 3D4/21 cells infected with SVA

**Figure 4:**
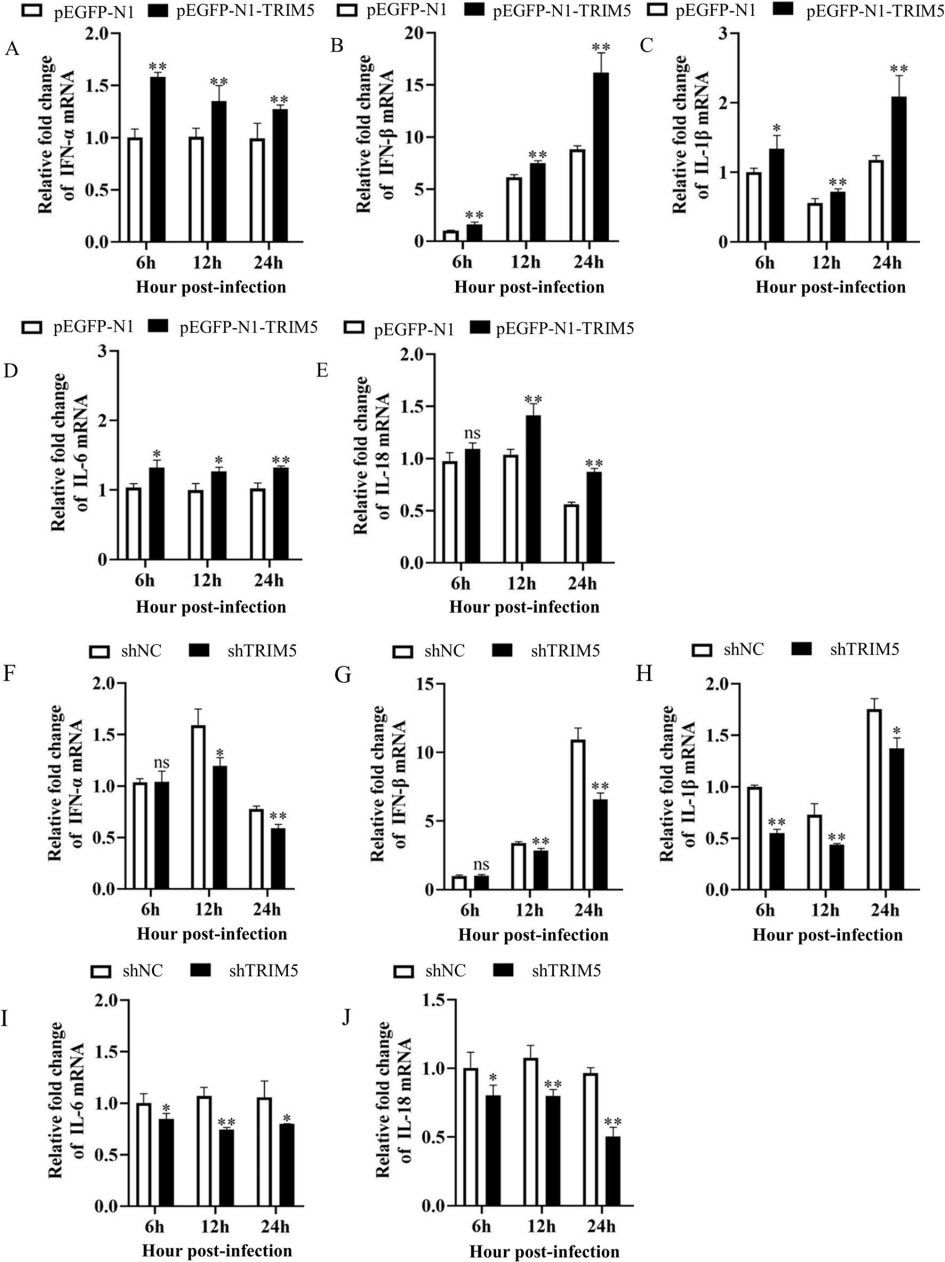
**TRIM5 increases the expression of IFNs and proinflammatory cytokines in 3D4/21 cells infected with SVA. A–E** 3D4/21 cells were transfected with pEGFP-N1 or pEGFP-N1-TRIM5 for 24 h and infected with SVA at an MOI of 1. The expression of IFN-α, IFN-β, IL-1β, IL-6, and IL-18 at 6, 12, and 24 hpi was measured by qRT-PCR. **F–J** 3D4/21 cells were transfected with shNC or shTRIM5 for 24 h and infected with SVA at an MOI of 1. The expression of IFN-α, IFN-β, IL-1β, IL-6, and IL-18 at 6, 12, and 24 hpi was measured by qRT-PCR. All experiments were repeated at least three times independently. ^*^P < 0.05, ^**^P < 0.01, ns indicates no significant difference.

SVA infection induces the expression of IFNs and proinflammatory cytokines, which participate in the pathogenesis of the virus. To determine whether TRIM5 regulates IFNs and proinflammatory cytokines in SVA-infected cells, pEGFP-N1-TRIM5 and shTRIM5 were transfected into 3D4/21 cells, which were subsequently infected with SVA. Viral infection experiments in TRIM5-overexpressing cells revealed that TRIM5 overexpression significantly increased the expression of IFN-α and IFN-β from 6 to 24 hpi (*P* < 0.01). IL-1β was significantly increased at 6 (*P* < 0.05), 12, and 24 hpi (*P* < 0.01); IL-6 was significantly increased at 6, 12 (*P* < 0.05), and 24 hpi (*P* < 0.01); and IL-18 was significantly increased at 12 and 24 hpi (*P* < 0.01) (Figure 4A–E). Moreover, viral infection experiments in cells with impeded expression of TRIM5 revealed that the expression of IFN-α at 12 (*P* < 0.05) and 24 hpi (*P* < 0.01); the expression of IFN-β at 12 and 24 hpi (*P* < 0.01); the expression of IL-1β at 6, 12 (*P* < 0.01), and 24 hpi (*P* < 0.05); the expression of IL-6 at 6 (*P* < 0.05), 12 (*P* < 0.01), and 24 hpi (*P* < 0.05); and the expression of IL-18 at 6 (*P* < 0.05), 12, and 24 hpi (*P* < 0.01) significantly decreased (Figure 4F–J). These results indicate that TRIM5 promotes the expression of IFNs and proinflammatory cytokines in SVA infection.

### TRIM5 promotes the expression of the RIG-I signalling pathway

**Figure 5:**
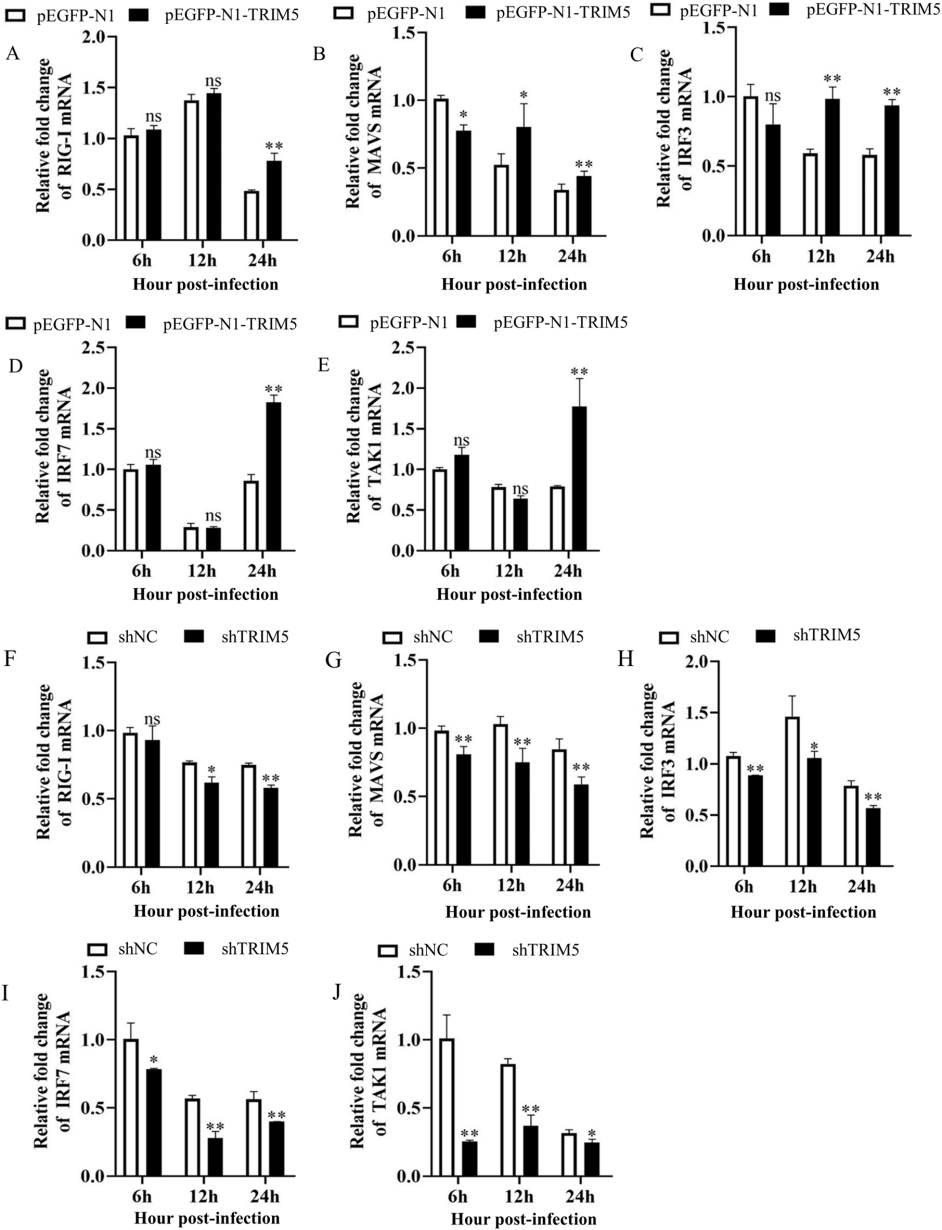
**TRIM5 promotes the expression of the RIG-I signalling pathway. A–E** 3D4/21 cells were transfected with pEGFP-N1 or pEGFP-N1-TRIM5 for 24 h and infected with SVA at an MOI of 1. Cells were collected at 6, 12, and 24 hpi, and the expression of RIG-I, MAVS, IRF3, IRF7, and TAK1 was measured by qRT-PCR. **F–J** 3D4/21 cells were transfected with shNC or shTRIM5 for 24 h and infected with SVA at an MOI of 1. Cells were collected at 6, 12, and 24 hpi, and the expression of RIG-I, MAVS, IRF3, IRF7, and TAK1 was detected via qRT-PCR. All experiments were repeated at least three times independently. ^*^P < 0.05, ^**^P < 0.01, ns indicates no significant difference.

Viral infection experiments were conducted on 3D4/21 cells with overexpression or inhibited expression of TRIM5 to determine whether TRIM5 regulates the expression of cytokines related to the RIG-I-like receptor signalling pathway during SVA infection. Viral infection experiments in cells overexpressing TRIM5 revealed that the expression levels of RIG-I at 24 hpi (*P* < 0.01); of mitochondrial antiviral signalling protein (MAVS) at 6, 12 (*P* < 0.05), and 24 hpi (*P* < 0.01); of interferon regulatory factor 3 (IRF3) at 12 and 24 hpi (*P* < 0.01); of IRF7 at 24 hpi (*P* < 0.01); and of TAK1 at 24 hpi (*P* < 0.01) were significantly increased (Figure 5A–E). Conversely, viral infection experiments in cells with impeded expression of TRIM5 revealed that RIG-I expression at 12 (*P* < 0.05) and 24 hpi (*P* < 0.01); MAVS expression at 6, 12, and 24 hpi (*P* < 0.01); IRF3 expression at 6 (*P* < 0.01), 12 (*P* < 0.05), and 24 hpi (*P* < 0.01); IRF7 expression at 6 (*P* < 0.05), 12, and 24 hpi (*P* < 0.01); and TAK1 expression at 6, 12 (*P* < 0.01), and 24 hpi (*P* < 0.05) were significantly lower than those in the control (Figure 5F–J). These results indicate that TRIM5 promotes the expression of IFNs and proinflammatory cytokines associated with the RIG-I-like receptor signalling pathway during SVA infection.

### TRIM5 promotes the expression of RIG-I, RIG-I-mediated IFNs and proinflammatory cytokines

**Figure 6:**
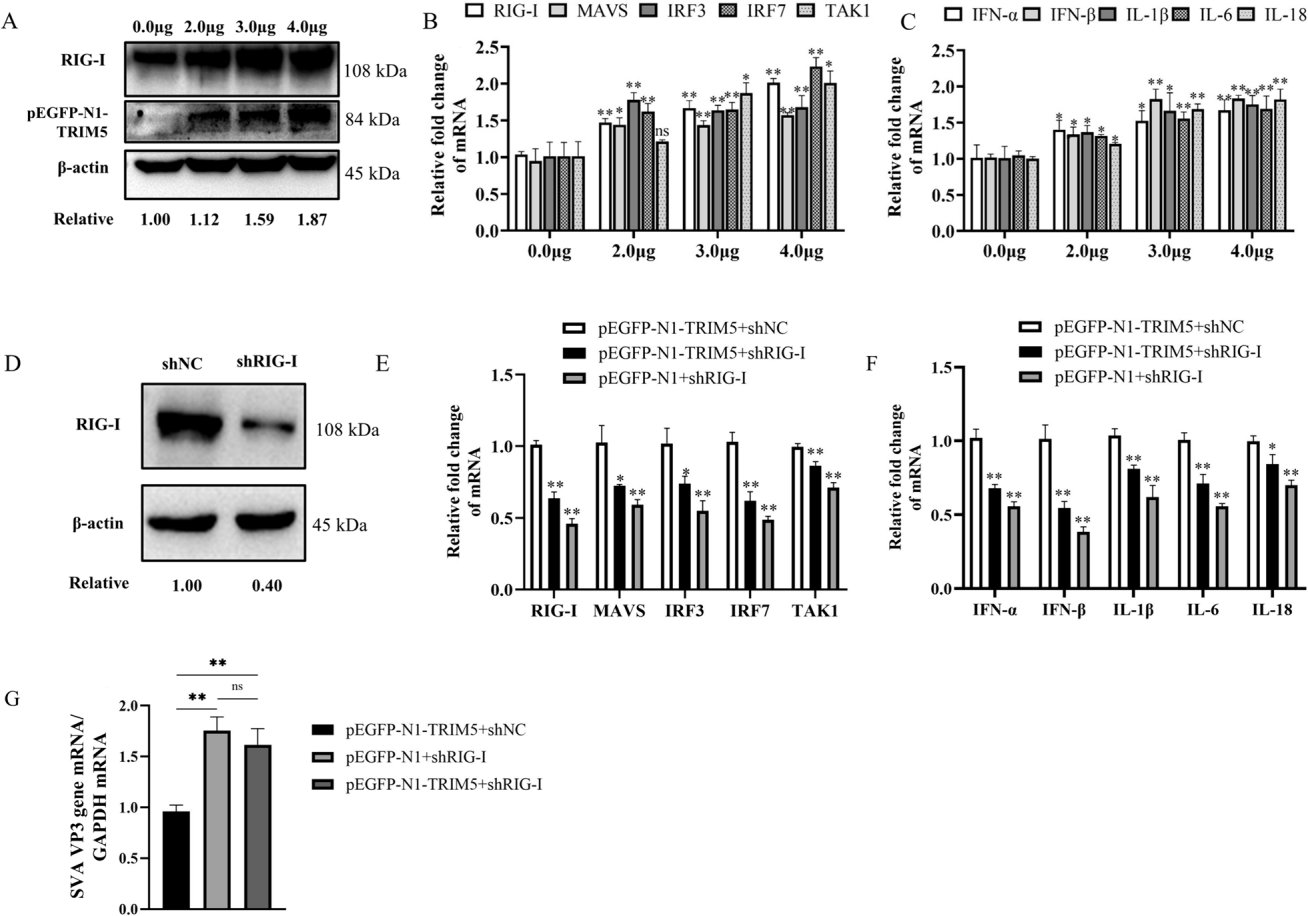
**TRIM5 promotes the expression of RIG-I and RIG-I-mediated IFNs and proinflammatory cytokines. A** 3D4/21 cells were transfected with different doses of pEGFP-N1-TRIM5, and the protein expression of RIG-I was detected using western blotting. **B**,** C** 3D4/21 cells were transfected with different doses of pEGFP-N1-TRIM5 and infected with SVA at an MOI of 1. The expression of RIG-I, MAVS, IRF3, IRF7, TAK1, IFN-α, IFN-β, IL-1β, IL-6, and IL-18 was measured by qRT-PCR. **D** The effectiveness of shRIG-I was detected by western blotting. **E**,** F** 3D4/21 cells were transfected with pEGFP-N1-TRIM5 and shNC, pEGFP-N1-TRIM5 and shRIG-I, or pEGFP-N1 and shRIG-I and infected with SVA at an MOI of 1. The expression of RIG-I, MAVS, IRF3, IRF7, TAK1, IFN-α, IFN-β, IL-1β, IL-6, and IL-18 was measured by qRT-PCR. **G** The mRNA expression of the SVA VP3 gene in 3D4/21 cells was measured by qRT-PCR. The experiment was repeated at least three times independently. ^*^P < 0.05, ^**^P < 0.01, ns indicates no significant difference.

To verify whether TRIM5 influences the expression of RIG-I, 3D4/21 cells were transfected with different doses of pEGFP-N1-TRIM5. The protein expression levels of RIG-I gradually increased with increasing doses of pEGFP-N1-TRIM5, indicating that the expression of RIG-I was promoted by TRIM5 (Figure 6A). Viral infection experiments in cells overexpressing TRIM5 revealed that RIG-I, IRF3, and IRF7 were expressed at concentrations ranging from 2 to 4 µg (*P* < 0.01); MAVS was expressed at 2 (*P* < 0.05), 3, and 4 µg (*P* < 0.01); TAK1 was expressed at 3 and 4 µg (*P* < 0.05); IFN-α was expressed at 2, 3 (*P* < 0.05), and 4 µg (*P* < 0.01); IFN-β was expressed at 2 (*P* < 0.05), 3, and 4 µg (*P* < 0.01); IL-1β was expressed at 2, 3 (*P* < 0.05), and 4 µg (*P* < 0.01); IL-6 was expressed at 2 (*P* < 0.05), 3, and 4 µg (*P* < 0.01); and IL-18 was expressed at 2 (*P* < 0.05), 3, and 4 µg (*P* < 0.01) (Figure 6B and C).

To explore whether TRIM5 promotes the IFN signalling pathway via RIG-I, 3D4/21 cells with inhibited RIG-I expression were generated and used for subsequent experiments (Figure 6D). Viral infection experiments involving RIG-I interference and TRIM5 overexpression in cells revealed that the expression of RIG-I (*P* < 0.01), MAVS (*P* < 0.05), IRF3 (*P* < 0.05), IRF7 (*P* < 0.01), TAK1 (*P* < 0.05), IFN-α (*P* < 0.01), IFN-β (*P* < 0.01), IL-1β (*P* < 0.05), IL-6 (*P* < 0.01), and IL-18 (*P* < 0.05) was decreased compared to that in the control group but was greater than that in the pEGFP-N1 + shRIG-I group (Figure 6E and F). Viral infection experiments showed that the mRNA expression of the SVA VP3 gene did not significantly differ between the pEGFP-N1 + shRIG-I and pEGFP-N1-TRIM5 + shRIG-I groups, indicating that TRIM5 regulates the RIG-I signalling pathway (Figure 6G). In summary, TRIM5 regulates the expression of RIG-I and promotes the expression of related molecules downstream of the RIG-I pathway.

## Discussion

SVA establishes persistent infection in swine herds and becomes an infection source, which makes it difficult to prevent and control in pig farms and has caused a worldwide outbreak of SVA-induced PIVD [9]. Host factors play a role in the response to viral infection, but their role in the replication of SVA has not been well defined [27]. As a virus-limiting factor that has been reported in HIV infection, TRIM5 works primarily by activating the innate immunity of host cells [23, 24]. Most recently, TRIM5 was reported to restrict the infection and replication of poxvirus [28]. Here, TRIM5 had a similar effect on limiting the proliferation of SVA cells.

The antiviral response of type I IFNs and proinflammatory cytokines is often inhibited in SVA infection [29, 30]. Our results showed that TRIM5 inhibits SVA replication by promoting the expression of type I IFNs (IFN-α and IFN-β) and proinflammatory cytokines (IL-1β, IL-6, and IL-18), which enhance the antiviral capabilities of the host. This finding is consistent with previous studies showing that TRIM family proteins inhibit virus replication by promoting the expression of type I IFNs and proinflammatory factors [31]. In addition, TRIM5 affects the entry of SVA, which is one of the antiviral roles of this factor; TRIM5α restricts HIV entry by binding to the incoming retroviral capsid (CA) protein, resulting in premature disassembly of the CA [32]; however, how TRIM5 regulates the entry of SVA and the detailed underlying mechanism need further research.

Previous studies have shown that TRIM5 can act as a signal transduction protein to initiate innate immune responses by activating the NF-κB and AP-1 signalling pathways [23]. We found that the inhibitory effect of TRIM5 on SVA relies on the production of RIG-I-mediated IFNs and that TRIM5 promotes the protein expression of RIG-I. After interfering with RIG-I proteins, the promotional effect of TRIM5 on RIG-I-mediated IFN-α and IFN-β decreased, and the inhibitory effect of TRIM5 on SVA replication did not counteract the promoting effect of interfering with the RIG-I protein, indicating that the anti-SVA effect of TRIM5 is mediated by RIG-I. A previous report showed that overexpression of RIG-I inhibits the proliferation of SVA, which is corroborative evidence of the antiviral effect of RIG-I on SVA infection in this study [33]. TRIM5 promoted the expression of RIG-I, which is consistent with the findings that TRIM4 and TRIM25 are both positive regulators of RIG-I-mediated IFN induction in the TRIM family [34]. The results shown here indicate that TRIM5 is essential for RIG-I-mediated IFN production and antiviral activity in response to SVA, which provides new insights into the regulatory effect of TRIM5 on the innate immune system.

In conclusion, TRIM5 inhibits the replication of SVA via RIG-I-mediated type I IFN antiviral response. These findings provide new data on host factors limiting the replication of SVA and indicate that targeting TRIM5 can aid in the development of new agents against SVA.

## Data Availability

The datasets generated during and/or analysed during the current study are available from the corresponding author upon reasonable request.
